# Paths towards Family‐friendly Working Time Arrangements: Comparing Workplaces in Different Countries and Industries

**DOI:** 10.1111/spol.12270

**Published:** 2016-10-26

**Authors:** Tobias Wiß

**Affiliations:** ^1^ Hertie School of Governance Berlin Germany

**Keywords:** Family policy, Industrial relations, Working time, Work–life balance

## Abstract

Although studies have examined the distribution and conditions of employer‐provided work–family arrangements, we still lack a systematic investigation of how these vary for different countries and industries. Based on the European Working Conditions Survey 2010, this study examines the conditions under which firms provide family‐friendly working time arrangements and what the differences are across four countries (Austria, Denmark, Italy and the UK) and four industries. The impact of employee representatives, employee involvement, manager support and female managers varies across countries and industries because of the institutional environment (prevailing family model, industrial relations) and workforce composition (gender). The impact of employee representatives depends on their co‐determination rights, and the direction of their effect on the prevailing family model (e.g. negative in conservative countries such as Austria) and the gender composition of the workforce (negative in male‐dominated production, but positive in services). Employee involvement in the work organization is significantly positive in Austria and Denmark (both with co‐operative industrial relations), while manager support has the strongest effect in the UK (liberal regime). At the industry level, female supervisors are positively associated with family‐friendly working time arrangements only in the male‐dominated production industry. These findings suggest that the effects of agency variables and their direction vary depending on the institutional context.

## Introduction

In line with the social investment paradigm (Esping‐Andersen *et al.* 2002; Morel *et al.* 2012), family policy addresses the reconciliation of work and family life. The focus of the debate has been mainly on public family policies, but family‐friendly working conditions depend largely on firm‐level policies (similarly, see Evans 2002). Family‐friendly working time arrangements increase work/family reconciliation and are an instrument to extend female employment rates and increase financial resources for households with children (Wood *et al.* 2003).^1^

Patterns of work/family reconciliation differ across firms or industries, because legislation imposes only minimum standards. The provision of and access to work–family policies often depend on the employer or manager and on collective negotiations. Not all flexible working time arrangements facilitate the combination of work and family life. This article refers to employee‐friendly working time arrangements (e.g. the possibility to take hours off for family reasons) and not to employer‐friendly working time flexibility (e.g. shift work) (for details, see Chung and Tijdens 2013; Gregory and Milner 2009).

Although studies have examined the distribution and conditions of work–family arrangements, we still lack a systematic investigation of how these vary for different countries and industries. Therefore, this article contributes to the literature by answering first, under what conditions employees have access to firm‐level family‐friendly policies, and second, what differences exist across countries and industries. The majority of studies focus either on single countries (Budd and Mumford 2004; Heywood and Jirjahn 2009; Ravenswood and Markey 2011; Rigby and O'Brien‐Smith 2010; Wood *et al.* 2003) or analyze a large number of countries and industries together (Chung 2008, 2009; Den Dulk *et al.* 2012; Evans 2002). In the first case, we cannot know whether the results hold true for other countries, and in the second case, we only know the average effect estimated for all countries. Studies across countries are well advised to stress country differences and not just their similarities and average effects (Mahoney and Goertz 2006). Not all conditions for firm‐level family policies are necessarily the same in all countries and industries; instead, some depend on the institutional environment specific to each country and industry, such as the system of industrial relations, and on the composition of the industry's workforce (e.g. gender and skill level) (for a similar argument, see Berg *et al.* 2004). Several authors argue that occupational welfare is conditioned by the institutional context (Den Dulk *et al.* 2012; Seeleib‐Kaiser and Fleckenstein 2009; for occupational pensions, see Ebbinghaus 2011). It is important to know whether factors that facilitate family‐friendly working time arrangements differ across countries and industries, because this can help reveal tailored strategies for advancing family‐friendly workplaces. Whether, for example, the existence of employee representatives or female managers increases the access to family‐friendly working time arrangements in one country or industry only but not in the others has implications for future family policies. Here, we analyze Austria, Denmark, Italy and the UK because they show important differences in welfare and family policies, working times and industrial relations systems, which may explain differences in the provision of family‐friendly working time arrangements. At the industry level, production, high‐value services, accommodation and trading activities, and public administration account for differences in the workforce regarding gender composition and flexibility requirements. Due to institutional variety in our countries and industries, we expect to find differences in firm‐provided family‐friendly working time arrangements.

Complementing studies based on company surveys, we reassess previous findings with regard to the conditions of family‐friendly working time arrangements and focus on their access at the individual level. This is important because firms might provide arrangements only to certain employees. The European Working Conditions Survey 2010 (EWCS) (the fifth such survey) from the European Foundation for the Improvement of Living and Working Conditions (Eurofound 2010a) examines flexitime and the option to take hours off for family reasons. Furthermore, in contrast to most company surveys, which cover only larger firms with at least ten employees, the EWCS also captures micro firms.

The following section reviews the main factors associated with firm‐level family policy including country and industry variation. Then, we present the data, variables and methods. Lastly, descriptive results offer an insight into the access to various time arrangements by country and industry. Based on logistic regressions, the fifth section analyses factors associated with family‐friendly working time arrangements, and whether they vary across countries and industries.

## Explaining Family‐friendly Working Time Arrangements Set at Firm Level

### Structural factors and agency

This article defines family‐friendly working time policies as arrangements that support employees in combining work and family life. Following the literature (Chung 2009; Seeleib‐Kaiser and Fleckenstein 2009), we refer to structural factors and agency to derive hypotheses. The article focuses especially on agency variables with regard to country and industry differences.

With regard to structural factors, previous studies assume that the skill composition of the workforce is important in determining employers' social policy preferences (Estevez‐Abe *et al.* 2001; Fleckenstein and Seeleib‐Kaiser 2011; Iversen 2005), distinguishing among employees with high general, specific and low general skills. Similar to the arguments developed for public social policies, employers might offer additional benefits, such as family‐friendly working time arrangements, to motivate and reward employees who invest in firm‐specific skills. Employees with firm‐specific skills are faced with higher (long‐term) unemployment risks because it is difficult to find a new job that requires the same firm‐specific skills. In contrast, one could argue that family benefits target female employees and not specifically qualified employees who are mainly male. Due to their firm‐specific skills, they are already committed to the firm. The portability of general skills could be one reason why employers offer work–family arrangements. Employees with high general skills are very important for firms, so workplace benefits are helpful instruments (Fleckenstein and Seeleib‐Kaiser 2011) to attract and keep them in competition with other firms. In addition, employees with high general skills such as professional staff are in a powerful position to ask for additional benefits (Evans 2002; Ravenswood and Markey 2011; Wood *et al.* 2003). In sum, we expect employers to provide family‐friendly working time arrangements more often to employees with high general skills and less often to employees with specific and low general skills (Hypothesis 1).

The size of a firm is considered as a further explanation for employer‐provided welfare (Bridgen and Meyer 2005; Den Dulk *et al.* 2012; Evans 2002; Mares 2001). Owing to economies of scale, large firms with substantial professional human resource departments are more likely to provide family‐friendly workplace policies (Hypothesis 2).

Actors and their interests and choices also influence work–family policies. Employees, their representatives, and trade unions can influence the introduction and provision of workplace family policies in bargaining agreements (Berg *et al.* 2014; Seeleib‐Kaiser and Fleckenstein 2009). Therefore, we can expect more family‐friendly workplace policies in firms and industries with strong trade unions or where employee representatives are able to negotiate favourable conditions for employees (Budd and Mumford 2004; Fleckenstein and Seeleib‐Kaiser 2011; Gregory and Milner 2009; Ravenswood and Markey 2011) (Hypothesis 3a). Beyond establishing family‐friendly working time arrangements, employee representatives can inform employees about existing policies and agreements (Budd and Mumford 2004). However, the access to and awareness of such policies and agreements by employees does not necessarily depend only on employee representatives. More generally, in some firms employees can directly participate in improving their working conditions when they have jobs with discretion or when the employer considers their ideas and suggestions for management decisions, resulting in an enhanced awareness of employees' needs (Wood *et al*. 2003). When employees are regularly involved in the organization of work and processes of the firm, and when they actively participate in improvements, they can attempt to implement family‐friendly work arrangements that are in their own interests (Hypothesis 3b).

Work–life balance policies can be in the interest of both employees and employers (Rigby and O'Brien‐Smith 2010). A business case is a factor when benefits for the employer outweigh the costs, resulting in better employee performances, less brain drain and a higher reputation (Den Dulk 2001; Den Dulk *et al.* 2012; European Commission 2009). For employers, the most important motives for providing family‐friendly working time arrangements lie in the recruitment and retention of qualified employees (Budd and Mumford 2004; Evans 2002; Fleckenstein and Seeleib‐Kaiser 2011; Wood *et al.* 2003). Furthermore, managers (for business reasons) might take care of their employees' needs and concerns, including those that involve work/family balance. Supporting managers who inform, consult and respect their employees are more likely to introduce family‐friendly working time arrangements (Wood *et al.* 2003) (Hypothesis 4).

Lastly, high percentages of female employees and female supervisors are considered to have a positive effect (Fleckenstein and Seeleib‐Kaiser 2011; Wood *et al.* 2003). In terms of female managers, the findings are ambiguous (for an overview, see Fleckenstein and Seeleib‐Kaiser 2011). From a theoretical point of view, one could argue that female managers share the needs and concerns of female employees and parents trying to combine work and family life (Hypothesis 5).

### Differences across countries

The relationship of some of the mentioned factors with family‐friendly working time arrangements is not necessarily the same in all countries (similarly, see Berg *et al.* 2004). Based on the institutional context and industrial relations, the effect of agency variables and their direction are likely to vary by country. Studies in the tradition of comparative capitalism (Amable 2003; Hall and Soskice 2001; Hancké *et al.* 2007) argue that capitalist economies are shaped by different institutional environments and mechanisms of coordination. In countries with strategic and non‐market coordination mechanisms (represented by Austria and Denmark), trade unions, high collective bargaining rates and employee representatives play a crucial role in promoting co‐operative industrial relations (for collective bargaining coverage and rights of employee representatives for all four countries, see Visser 2013). They are part of the social‐democratic (Denmark) or conservative (Austria) welfare regime (for welfare regimes, see Bonoli 1997; Ferrera 1996) with high support for the dual‐earner model, exhibiting work/family reconciliation in the former and high male breadwinner support in the latter (Den Dulk *et al*. 2012; Mischke 2011). Austria as a transitional working time regime (Figart and Mutari 2000) shows medium levels of gender equity. Also, employer‐friendly flexibility is more prevalent than employee‐friendly working time arrangements (Chung and Tijdens 2013). As a forerunner in family‐friendly employment policies, Denmark represents a solidaristic gender equity regime that supports the dual earner model with more employee‐friendly working time arrangements (Chung and Tijdens 2013; Figart and Mutari 2000).

Liberal countries such as the UK are characterized by market‐based coordination mechanisms with weak trade unions, low collective bargaining levels and employee representatives with limited rights. Family issues are viewed primarily as a private responsibility in liberal welfare regimes, and public support for dual earners is rather low (Mischke 2011). Compared with Denmark, the UK is a liberal flexibilization regime that shows a lower level of gender equity. Employer‐friendly working time arrangements are common (Chung and Tijdens 2013; Figart and Mutari 2000). In Italy, which represents a Mediterranean welfare and male breadwinner regime (Figart and Mutari 2000), collective bargaining coverage is high, but employee representatives are weak. Public support for the dual earner model is moderate because of financial constraints (Mischke 2011), and the levels of gender equity and working time flexibility for both employers and employees are low (Chung and Tijdens 2013; Figart and Mutari 2000).

These structural differences, together with disparities in the level of the historical development of family‐friendly policies (with Denmark as a forerunner and Italy and Austria as laggards), have implications for both the scope and some conditions of firm‐provided family‐friendly working time arrangements. The institutional context creates a normative climate in which firm‐level family policies conform to public family policies and societal norms (Den Dulk *et al.* 2012).

Although we test the relation of several factors with family‐friendly working time arrangements, (theoretical) support for country variations are evident only for some of them. We expect that the role of employee representatives, manager support and employee involvement vary among countries for the following reasons. Although employee representatives are equipped with co‐determination rights in Austria, we do not expect a strong positive effect on work–family policies because the prevailing male breadwinner model is the ideal family form in conservative countries. This would be in line with the general argument of Ravenswood and Markey (2011) that family‐friendly policy is not seen as relevant for unions in male‐dominated environments (or with men as the dominant breadwinners in our case). In contrast, the strong employee representation in family‐friendly Denmark should have a positive impact because it can effectively improve the combination of work and family life within the context of widely accepted working mothers and fathers. The weak employee representation in Italy, embedded in a Mediterranean environment with men as the dominating breadwinner, is probably less suited to promote family‐friendly working time arrangements. We also expect that employee representatives' limited rights in the UK (Visser 2013) mean they will have no significant effect. From this derives a more important role for the employer and manager in the UK, because decisions are made mainly at the firm level at the discretion of the employer (similarly for liberal welfare states such as the USA and Australia, see Berg *et al.* 2004). The family‐friendly climate in Denmark and the support of the dual earner model can also affect employers. Normative pressure could produce conformity (DiMaggio and Powell 1983) resulting in a similar positive effect of managers on family‐friendly working times in line with societal beliefs about work/family reconciliation. We do not expect that managers contribute to work/family reconciliation in Austria and Italy, because of the dominance of the male breadwinner model in those countries. With regard to the individual involvement of employees, the positive effect should be stronger in Austria and Denmark compared with Italy and the UK, because the industrial relation systems and management‐employee relations are more co‐operative and coordinated in the former countries, resulting in employers who recognize and appreciate employee participation avoiding conflicts.

### Differences across industries

Irrespective of their country of location, industries matter when considering variations in work/family policies and can be even more important than (welfare) state characteristics (Den Dulk *et al.* 2012). Industry‐specific circumstances such as the composition of the workforce and flexibility requirements – the situational perspective (Wood *et al*. 2003) – affect family‐friendly practices and can result in different effects for agency variables such as employee representatives and managers. The production industry, with many specifically qualified employees, shows a low operational flexibility (for flexibility profiles, see Eurofound 2010b). In contrast to other industries, male employees – who account for more than two‐thirds of the workforce – are dominant and the number of female managers is very low (for the workforce composition of all industries, see Appendix table A2). As a role model for gender equality, the public sector (Den Dulk *et al.* 2012) is considered to be more family‐friendly than the private sector (European Commission 2009). Public administration exhibits average levels of various forms of working time flexibility, and when compared with the private sector, trade unions are stronger and employee representatives are more common.

Among private services that require more flexibility from employees than the production industry (European Commission 2009), this article analyses high‐value services such as the financial industry, which has very high operational working time flexibility and a high share of female employees. Firms in accommodation and trading activities also offer high flexibility, but mainly to cope with long opening hours. In addition, most of their employees possess low general skills. The share from small firms and part‐time employees is very high, and employee representatives are not very common.

Similar to the country level, arguments and literature support variation among industries especially for the role of employee representatives, manager support and female managers (Berg *et al.* 2004). We expect a positive effect for employee representatives in all industries except production. Although employee representatives are usually common in the production industry, which has many large firms, we expect them to represent mainly the interests of male employees, because women are in the minority, and therefore demands for family‐friendly working conditions are weak (Ravenswood and Markey 2011). In terms of manager support, we expect a negative or at least no significant positive relation for manager support in high‐value services such as financing. Based on findings of Den Dulk *et al.* (2011) for the financial sector, it is very likely that the performance of a department and operational needs are threatened by the disruptiveness of work–life balance policy in this sector. In contrast, female managers probably exert a particularly positive influence in the production industry, where they are not common. Where female managers exist in a male‐dominated environment, they both make a difference and (also) recognize the interests of women and parents and the importance of ‘female’ topics such as work/family reconciliation. We lack (theoretical) support for country and industry variation of other factors (e.g. skills and firm size), but for the sake of completeness we also test their variation.

## Empirical Strategy and Measures

### Data

The analyses presented here are based on the EWCS (Eurofound 2010a). The cross‐sectional dataset – carried out every five years with partly varying questions – provides representative information on the quality of work in 34 countries with, for 2010, 43,800 observations. Individuals aged 15 or over (16 or over in Spain, the UK and Norway) who are employed were interviewed face to face based on a random sampling design. The questionnaire allows studying family‐friendly working time arrangements together with individual and firm‐level characteristics. Because this article is interested in family‐friendly working time arrangements for employees provided by firms, self‐employed respondents, who have much more control over their working hours, are excluded from the analyses.

We tested the conditions for family‐friendly working time arrangements for theoretically selected different institutional contexts. All regressions were run separately for each country and industry (for sample statistics, see Appendix tables A1 and A2). Austria (n = 819), Denmark (n = 958), Italy (n = 1,102) and the UK (n = 1,333) represent different welfare regimes, industrial relations systems and working time regimes as described earlier.

The number of cases for industries does not allow running our models separately for each industry within any single country (e.g. production in Austria). For this reason, we include the EU‐15 countries for the industry‐models and control for countries, allowing the analysis of industries without country effects.^2^ We merged the industries based on NACE Rev. 2 to increase the number of observations, resulting in: production with 2,663 observations (mining, manufacturing, electricity and water supply), high‐value services with 1,494 observations (financial, real estate and professional activities), accommodation and trading activities with 3,746 observations (wholesale, retail, accommodation and food services) and public administration with 1,334 observations. These four industries cover different workforce compositions, as shown above, and account for the public and private sectors as well as the industrial and service sectors.

### Dependent variables

The two dependent variables are flexitime and taking hours off. Here, flexitime applies when employees are responsible for determining their working hours, either entirely or within certain limits, by themselves. The determination of working hours by employees or flexitime results in higher working time sovereignty, thus enabling work–life balance (Possenriede and Plantenga 2014; Russell *et al.* 2009). The variable taking hours off is operationalized as an arrangement to take an hour or two off during working hours to take care of personal or family matters. It is coded as 1 when it is not or not too difficult and as 0 when it is somewhat or very difficult. This variable measures working time flexibility during work, which is important for work/family reconciliation in the case of unforeseeable family matters.

### Independent variables

In line with the theoretical considerations and previous studies, we consider independent variables measuring structural factors and agency. In terms of agency, the existence of an employee representative (dichotomous) and employee involvement in improving the work organization or work processes of the department or organization (answers ranging from 1 always to 5 never) are included as proxies for organized labour and employee involvement. Regarding manager support, individuals were asked if their manager helps and supports them (answers ranging from 1 always to 5 never). The question, ‘Is your immediate boss a man or woman?’ measures whether a supervisor is female.

The variable ‘employee skills’ was constructed according to the international classification systems for occupation (ISCO‐08). Employees are equipped either with high general (managers, technicians and associate professionals), specific (craft workers or machine operators, and assemblers) or low general skills (clerical support, service workers and elementary occupations) (similarly, see Estevez‐Abe *et al.* 2001; Fleckenstein *et al.* 2011; Seeleib‐Kaiser *et al.* 2012). The size of the firm is included in five categories (1–9, 10–49, 50–99, 100–499, and 500+ employees). For the country‐level regressions, nine dummy variables measure industries (based on NACE Rev. 2), and the industry‐level regressions control for 15 countries.

Gender, age, children aged 0–5 in the household, and part‐time work (≤ 34 working hours per week) control for individual characteristics. Net income (earnings from the main job) is measured with four categories constructed separately for each country and industry to account for differences in prices and purchasing power.

### Method

This article first shows descriptively the unequal distribution of family‐friendly working time arrangements (flexitime and taking hours off) across countries and industries. Then it describes the logistic regression models applied separately for each country, industry, and outcome variable to identify factors associated with family‐friendly working time arrangements. Dependent variables are dichotomized to run binary logistic regression models, because they are less complex and more intuitive to interpret compared with ordered logit models. The results are reported as average marginal effects showing the direction of associations and standardized effect sizes in percentages (Mood 2010). In terms of multicollinearity, the variance inflation factor is below five for all variables in all models, and therefore much below critical values greater than ten that would signal collinearity problems. The study uses two pooled models, one with all four countries and one with all four industries, along with a full interactions test to identify significant differences of the conditions across countries and industries, respectively (available from the author upon request). Table 2 (results by country) also shows the results for a model with all 34 country of the dataset and table 3 (results by industry) also shows the results for a model with all industries to ease the interpretation of country/industry differences.

## Prevalence of Family‐friendly Working Time Arrangements in Four Countries and Four Industries

The public working time legislation grants parents with children in Austria the right to request part‐time work (only in firms with more than 20 employees) and two weeks paid leave per year to care for ill children (Rille‐Pfeiffer and Dearing 2014). In Italy and the UK, employees can apply for reduced working hours, but employers can refuse it for serious business reasons. In addition, in the UK and Italy, employees can take time off (unpaid) to address dependant emergencies, such as to go the doctor if a child falls ill (Addabbo *et al.* 2014; O'Brien *et al.* 2014). Due to these minimum standards (that mainly concern part‐time or permanent reduced work or emergency situations), there is room to manoeuvre at the firm level and for (collective) agreements to set family‐friendly working time arrangements. In Denmark, for example, the right to take a day off (paid) to care for a sick child is included in most of the working contracts and collective agreements (Bloksgaard and Rostgaard 2014).

Denmark ranks first on both measures of family‐friendly working time arrangements (see table 1). In detail, almost half the employees in Denmark can use flexitime compared with only 31 per cent in Austria, 22 per cent in the UK and 17 per cent in Italy. The possibility to take one or two hours off to take care of family matter is lowest in Austria (70 per cent), followed by the UK and Italy (each 74 per cent). In contrast, employees benefit from this firm‐level family policy much more in Denmark (82 per cent).

**Table 1:** Prevalence of family‐friendly working time arrangements (% employees)

Countries	AT	DK	IT	UK
Flexitime	31.3	48.4	17.1	22.2
Taking hours off	69.7	81.6	73.9	73.5

Industries	Production	High‐value services	Public administration	Accommodation trading
Flexitime	26.6	44.8	33.3	21.1
Taking hours off	72.1	74.9	76.7	62.3

*Source*: EWCS (Eurofound 2010a), weighted results.

At the industry level, public administration and high‐value services show the highest levels of family‐friendly working time arrangements and accommodation and retail show the lowest (see table 1). In the financial and real estate industry, 45 per cent of employees can use flexitime, followed by public administration (33 per cent), and production (27 per cent). Only 62 per cent of employees in accommodation and retail and wholesale have the possibility to take hours off for family reasons compared with 77 per cent in public administration and 75 per cent in high‐value services.

## The Determinants of Family‐friendly Working Time Arrangements

### Countries

In line with hypothesis 1, employee skills are important for the setting of working time arrangements and partially for the possibility to take hours off (see table 2). Employees with low general and specific skills have less discretion over their working hours (flexitime) than employees with high general skills. Employees with specific skills are also less likely to take hours off compared to employees with general skills in Denmark and Britain. In line with Seeleib‐Kaiser and Fleckenstein (2009), who looked at the availability of firm‐level family polices, employers offer family‐friendly working time arrangements to employees to attract and retain those with general skills rather than to motivate investments in specific skills.

**Table 2:** Logit models by country for the conditions of family‐friendly working time arrangements (average marginal effects)

	Flexitime					Taking hours off				
	All 34 countries	AT	DK	IT	UK	All 34 countries	AT	DK	IT	UK
Employee representative	‐0.02***	‐0.06	‐0.12***	0.02	‐0.03	‐0.02***	‐0.07*	0.01	0.05	0.02
*Involvement in improving work organization (ref. never)*										
Rarely	0.03***	0.04	0.07	0.04	0.13**	0.02**	‐0.07	0.01	0.07	‐0.01
Sometimes	0.06***	0.08	0.09	0.03	0.11**	0.06***	0.03	0.08**	0.06	0.03
Most of the time	0.10***	0.12**	0.10	0.07	0.12**	0.10***	0.18***	0.12***	0.04	0.01
Always	0.13***	0.16***	0.12*	0.10*	0.18***	0.12***	0.10*	0.16***	‐0.03	0.03
*Manager support (ref. never)*										
Rarely	0.01	‐0.07	0.09	‐0.04	0.03	0.01	‐0.11	0.01	‐0.03	0.03
Sometimes	0.02	0.00	0.21	‐0.01	0.19	0.04***	‐0.03	0.08	0.00	0.14**
Most of the time	0.01	0.00	0.21	‐0.04	0.18	0.09***	0.00	0.12*	0.07	0.22***
Always	0.00	‐0.02	0.23*	0.02	0.17	0.16***	0.08	0.18**	0.10*	0.32***
Female supervisor	‐0.01*	‐0.01	0.03	0.02	0.01	‐0.01*	‐0.03	‐0.02	0.01	0.00
*Skills (ref. high general)*										
Specific	‐0.15***	‐0.25***	‐0.25***	‐0.08**	‐0.13***	‐0.08***	‐0.04	‐0.09*	0.00	‐0.11**
Low general	‐0.10***	‐0.15***	‐0.08**	‐0.04	‐0.16***	‐0.03***	0.03	0.01	0.02	‐0.05
*Firm size (ref. 1–9 employees)*										
10–49	‐0.04***	0.04	0.00	‐0.02	‐0.05	‐0.01*	‐0.02	0.04	0.03	‐0.06
50–99	‐0.02***	0.17***	0.04	0.02	‐0.03	‐0.03***	‐0.06	0.03	0.00	‐0.11**
100–499	0.00	0.06	0.05	0.02	‐0.06	‐0.03***	‐0.04	0.01	0.03	‐0.11**
500+	0.03***	0.21***	0.17**	0.03	‐0.02	‐0.03**	0.03	0.04	0.04	‐0.11**
Controls	yes^a^	yes	yes	yes	yes	yes^a^	yes	yes	yes	yes
n	28,962	692	890	841	1,046	28,846	688	883	844	1,041
Nagelkerke R^2^	.290	.269	.280	.092	.223	.157	.199	.259	.096	.139

*Source*: EWCS (Eurofound 2010a).*Notes*: Controls: industry, sex, age, children aged under six in the household, part‐time and income.

a = additional control for countries.

*** p < 0.01.

** p < 0.05.

* p < 0.1.

Firm size is, if at all, not linearly associated with family‐friendly working time arrangements rejecting hypothesis 2. Although very large firms with more than 500 employees in Austria and Denmark have a strong positive effect on flexitime, they are negatively related to taking hours off in the UK. Contradicting previous studies (e.g. Budd and Mumford 2004; Den Dulk *et al.* 2012), this results from the integration of very small firms in the EWCS, whereas in most of the other company surveys and data only firms with at least ten or even 100 employees are included. In very small firms, contacts between employer and employee(s) are sometimes familiar and intense, and the employer can directly determine whether family‐friendly arrangements fit both the employer and the employee. Furthermore, work–life balance offered by small firms can be a competitive advantage compared with large firms that offer higher salaries.

Analyzed across countries, employee representatives do not have significant positive associations with family‐friendly working time arrangements, rejecting hypothesis 3a. Although organized labour has a positive effect on the provision of employer‐provided family policies in the article of Seeleib‐Kaiser and Fleckenstein (2009), the results are in line with the study of Den Dulk *et al.* (2012), where the degree of unionization is negatively associated with flexible work arrangements, and with the study of Budd and Mumford (2004), where trade unions have a significant negative relationship with flexible working hours. Our findings in regard to individual data and the role of employee representatives complement these studies. Our expectations regarding country differences are confirmed for all four countries except Denmark. Employee representatives are negatively associated with flexitime in Denmark and taking hours off in Austria (the negative effect in both cases is much stronger compared with the 34‐country model). The results are not significant for Italy and the UK. In the pooled model with country interactions, employee representatives in Denmark (for flexitime) and Austria (for taking hours off) have a negative and significant different effect compared with Italy. The somewhat unexpected result for Denmark may be because employee representatives often represent different unions. A lack of consensus between different unions or employees at the firm level with regard to flexible working time arrangements may hamper firm‐level agreements (Ilsøe 2012). A coalition problem among employees arises, because often only some employees would profit from flexitime. In contrast to the other three countries, works councils in Austria might be sceptical about flexible working time arrangements, associating them only with employer‐friendly working times. In a regularly conducted survey of employee representatives in Austria, only 12 per cent reported that they dealt with family‐friendly working conditions in the last 12 months of 2010 and only 10 per cent with employee requests for flexible working hours (Gerhartinger *et al*. 2010). Among 21 topics, they are ranked 18th and 19th, respectively. Furthermore, company agreements about family‐friendly working conditions exist in only 7 per cent of Austrian firms with employee representatives, the lowest number among the 21 topics (Braun and Specht 2009).

In contrast to organized labour and in line with hypothesis 3b, employees are more likely to work in family‐friendly workplaces when they are involved in improving the work organization or processes. The results are positive and significant for all four countries for flexitime and also for Austria and Denmark for taking hours off, confirming our expectation about country differences. Even when employees participate in the improvement of the work organisation in Italy and the UK, participation does not increase their probability of taking hours off for family reasons. It seems that the work environment in Austrian and Danish firms, which is in line with their strong social partnerships and cooperative management‐employee relations, is more likely to take employees' concerns into account. The pooled model with full interactions confirms the country differences.

Manager support does not increase flexible working time arrangements, except for the very strong support in Denmark. Nevertheless, hypothesis 4 is partly confirmed. Employees with strong manager support or help have significantly better possibilities to take hours off, especially in Denmark and the UK. In Denmark, both employee involvement and manager support are positively related to family‐friendly working time arrangements due to the dominant dual earner model. Although manager support does not promote the use of flexitime in the UK, employees' chances to profit from the possibility to take hours off are 32 per cent higher in firms where they always receive support by their manager compared with firms without manager support, 22 per cent when they receive support most of the time and 14 per cent when they receive support sometimes. This indicates a strong reliance of employees on the manager for taking hours off in the UK. British managers may use it for motivation or strategy reasons.

### Industries

Similar to the country analyses and in line with hypothesis 1, employees with high general skills have the most discretion over their working hours and are more likely to have the possibility to take hours off in all industries (see table 3). The results of the models by industry also confirm the ambiguous effect of firm size from the country analyses.

**Table 3:** Logit models by industry for the conditions of family‐friendly working time arrangements (average marginal effects)

	Flexitime					Taking hours off				
	All industries	Production	High‐value services	Public adm.	Accomod., trading	All industries	Production	High‐value services	Public adm.	Accomod., trading
Employee representative	‐0.01*	‐0.03*	0.06**	0.07**	‐0.02	0.00	‐0.03*	‐0.02	0.03	0.00
*Involvement in improving work organization (ref. never)*										
Rarely	0.05***	0.09***	0.04	0.17***	0.03	0.04***	0.02	0.05	0.06	0.01
Sometimes	0.08***	0.11***	0.03	0.12**	0.09***	0.08***	0.06**	0.06*	0.08**	0.10***
Most of the time	0.13***	0.15***	0.10**	0.16***	0.11***	0.12***	0.10***	0.08**	0.08**	0.14***
Always	0.16***	0.13***	0.14***	0.18***	0.15***	0.14***	0.12***	0.09**	0.11***	0.14***
*Manager support (ref. never)*										
Rarely	0.01	0.01	‐0.06	0.07	0.06*	0.01	0.05	‐0.04	0.06	‐0.01
Sometimes	0.03**	0.07*	‐0.14**	0.08	0.07**	0.04***	0.03	‐0.04	0.08*	0.02
Most of the time	0.02*	0.05	‐0.03	0.09	0.06*	0.08***	0.13***	0.01	0.10**	0.09***
Always	0.01	0.08**	‐0.09*	0.06	0.02	0.17***	0.17***	0.07	0.14***	0.20***
Female supervisor	‐0.01	0.05**	‐0.03	0.04	‐0.03**	‐0.01	0.02	0.02	0.00	‐0.02
*Skills (ref. high general)*										
Specific	‐0.19***	‐0.26***	‐0.15	‐0.29***	‐0.14***	‐0.07***	‐0.03	‐0.21**	‐0.17***	‐0.04
Low general	‐0.12***	‐0.10***	‐0.12***	‐0.20***	‐0.16***	‐0.02*	0.00	‐0.02	‐0.10***	‐0.04*
*Firm size (ref. 1–9 employees)*										
10–49	‐0.02**	‐0.05*	‐0.01	0.09**	0.01	‐0.01	0.02	0.01	0.00	0.02
50–99	0.00	‐0.04	‐0.02	0.04	0.03	‐0.03**	0.02	‐0.06	‐0.01	‐0.03
100–499	0.03***	0.01	0.11***	0.11**	0.07***	‐0.02*	0.01	0.01	‐0.04	0.01
500+	0.06***	0.03	0.16***	0.16***	0.07*	0.00	0.01	0.03	‐0.05	0.09*
Controls	yes^a^	yes	yes	yes	yes	yes^a^	yes	yes	yes	yes
n	15,243	2,388	1,316	1,058	3,144	15,218	2,380	1,317	1,054	3,126
Nagelkerke R^2^	.287	.364	.364	.419	.238	.175	.224	.187	.159	.199

*Source*: EWCS (Eurofound 2010a).*Notes*: Controls: country, sex, age, children aged under six in the household, part‐time and income.

a = additional control for industries.

*** p < 0.01.

** p < 0.05.

* p < 0.1.

Employee representatives are negatively associated with both measures of family‐friendly working time arrangements only in the production industry. In contrast, employee representatives have a positive effect on flexitime in high‐value services and public administration. This quantitative finding confirms the case study of Ravenswood and Markey (2011). In the male‐dominated production industry, employee representatives mainly act on behalf of male employees, for whom work–life balance might be perceived as a women's issue and is therefore not a priority compared with other topics. In high‐value services and the public administration, employee representatives are faced with much higher levels of female employees for whom work/family reconciliation matters.

If managers in high‐value services regularly support their employees, the latter are less likely to have flexitime. Manager support seems not to facilitate work/family reconciliation, but rather other aspects, perhaps because many employees (45 per cent) can already make use of flexitime. According to Den Dulk *et al.* (2011), managers in the financial sector with long working hours do not exactly act as role models and their considerations about work–life balance are restricted by disruption concerns. In contrast, employees receiving managerial support always or most of the time are more likely to profit from the possibility to take hours off in production, public administration, and accommodation and trading.

Having a female supervisor significantly increases only the use of flexitime in the production industry. If a woman is in a managerial position within an otherwise male‐dominated environment, she improves work/family reconciliation thanks to her higher awareness of parents' needs and concerns. A possible reason for the negative effect of female supervisors for flexitime in accommodation and trading could be that female managers in this industry are more junior than senior managers and that they lack the power to influence work/family policies (Milliken *et al*. 1998). It could also be that those with female managers are more in low‐wage jobs. To our knowledge, there are no good reasons to justify industry variation for employee involvement. In fact, we find a linear positive relation for employee involvement in improving the work organization with family‐friendly working time arrangements in all industries.

## Conclusion

Family policy is a core element of the social investment paradigm. However, attention has been paid mainly to public family policies and differences between countries. It is necessary to also consider workplace‐driven family policies to capture the overall extent of work/family reconciliation. In this sense, family‐friendly working time arrangements are an important supplement to public family policies.

This study used data from the EWCS to analyze the conditions for family‐friendly working time arrangements and their variances across countries and industries due to different institutional environments. The descriptive statistics showed that the share of employees who entirely or partly set their working hours by themselves (flexitime) and for whom arranging to take hours off to take care of family matters is rated either not difficult at all or not difficult is highest in Denmark, public administration and financing.

With regard to the conditions for family‐friendly working time arrangements, several logit models tested hypotheses for structural factors and agency derived from previous studies. The results confirmed hypothesis 1 (skills), but not hypothesis 2 (firm size). Firms use work–family arrangements to attract and retain employees with high general and – at lower levels – low general skills in competitive labour markets, rather than to motivate investments in firm‐specific skills. In general, larger firms do not provide more family‐friendly working time arrangements than small firms. The findings of Budd and Mumford (2004), Den Dulk *et al.* (2012), and Seeleib‐Kaiser and Fleckenstein (2009), who focused mainly on the provision of firm‐level family policies and companies with more than ten employees, do not hold true when small firms are included. In some cases, employees benefit especially in very small firms from family‐friendly working time arrangements due to intense relations with the employer and a more familiar atmosphere.

Although skills show a similar relation with family‐friendly working time arrangements in all countries and industries (and firm size a very ambiguous relation), different institutional environments reflect varying relations for employee representatives (and partly employee involvement), manager support and female supervisors. Hypothesis 3a, concerning the positive effect of employee representatives, is partly rejected. As expected, employee representatives are significantly associated with family‐friendly working time arrangements in Austria and Denmark, but not in Italy and the UK, because they only have co‐determination rights in the first two countries. Their effect is negative in Austria due to the prevailing male breadwinner model in a conservative country and – unexpectedly – also negative in Denmark, probably because of different unions' presence within firms and coalitional problems. The lacking or weak association of employee representatives with family‐friendly working time arrangements in Italy and the UK is testimony to the extent to which their influence on firm policies has declined in recent years. It could also be the case that employers are not willing to engage meaningfully with (weak) employee representatives on this topic and that employers' obstruction prevents a positive effect. Therefore, there is a need for further research to explain whether the lacking or weak effect of employee representatives is due to employers' intransigence or to failings of employee representatives. In contrast to the institutionalized power of employees, their direct involvement in improving the work organization has a positive effect for both measures only in Austria and Denmark, where a strong social partnership and co‐operative management‐employee relations characterize the work environment.

With regard to industry differences, employee representatives have negative effects only in production, but their effects are partly positive in other industries. Employee representatives in the production industry may give priority to traditional bargaining areas such as wages, and be insensitive to ‘female’ areas such as gender and family issues (Berg *et al*. 2014) as they act on behalf of mainly male employees. In contrast, in the public and private services where there are more female employees, their representatives defend parents' interests.

We found only partial support for hypothesis 4. High manager support also increases the use of family‐friendly working time arrangements in Denmark thanks to the prevailing and accepted dual earner model in contrast, for example, to Austria. Manager support is the strongest condition for taking hours off in the UK and (without positive effects for employee representatives and employee participation) indicates a strong reliance of employees on the employer, typical for a liberal country.

Evidence for hypothesis 5 is very weak. Female supervisors improve the combination of work and family life only in the production industry, whereas no such effect exists for the other industries and countries. They may share the needs and concerns of female employees, providing an effect that is particularly noticeable in an otherwise male‐dominated environment. The main lesson from the variation across countries and industries is that the relation of employee representatives (and partly employee involvement), manager support and female supervisors with family‐friendly working time arrangements is not the same for all countries and industries. Strong employee representative alone, for example, are not a guarantee for work–life balance policies. The relation depends not only on their power, but also on the institutional environment such as, for example, prevailing norms about work–family reconciliation and working mothers. Future research should consider this.

As its main limitation, this article applies only to family‐friendly working time arrangements and not to other firm‐level family policies, because measures such as extra‐statutory leave programmes or employer‐provided childcare are not included in the EWCS. Future research may apply our design and test the findings for other firm‐level family practices. Although we show associations of certain conditions with family‐friendly working time arrangements, qualitative and longitudinal studies could complement our findings with detailed insights and causal mechanisms for the establishment and promotion of family policies within firms in varying institutional contexts.

In addition to the mere provision of family‐friendly workplace practices, their usage and awareness of all and not only some groups of employees calls for further attention and research. In terms of equality of men and women, and to increase the reconciliation of work and family life, we need more efforts to reduce variations across countries at European level and across industries at the country level.

## Notes

1 The article is only about a dimension of family‐friendly working, rather than the whole concept of work/family reconciliation.

2 We acknowledge that there may be variances of industries across countries due to data limitations.

## Appendix

**Table A1:** Sample statistics by country for the independent variables

		AT		DK		IT		UK	
	Range	Per cent/median	Std. dev.	Per cent/median	Std. dev.	Per cent/median	Std. dev.	Per cent/median	Std. dev.
Employee representative		45		76		38		51	
Involvement in improving work organization	1–5	2.81	1.45	3.67	1.25	3.03	1.41	3.12	1.52
Manager support	1–5	3.41	1.34	3.95	1.02	3.19	1.26	4	1.06
Female supervisor		28		36		25		42	
*Skills*									
High general skills		30		53		41		40	
Specific skills		22		16		18		12	
Low general skills		48		31		41		48	
*Firm size (no. employees)*									
1–9		30		19		37		22	
10–49		32		40		30		31	
50–99		11		15		12		12	
100–499		16		17		12		19	
500+		10		9		9		16	
*Industry*									
Production		20		20		19		10	
High‐value services		8		7		10		7	
Public administration		5		6		9		9	
Accommodation and wholesale/retail		27		13		19		22	
Other services		8		6		8		10	
Construction		7		5		5		5	
Education		7		12		12		11	
Health		10		23		8		17	
Other industries		8		9		10		9	
Female		56		51		53		55	
Age		41.62	11.61	43.01	12.56	41.44	10.79	41.38	12.5
Children <6 years		13		17		12		17	
Part‐time		29		28		29		40	
*Income*									
Low		36		44		42		28	
Middle		27		25		17		23	
High		24		29		15		20	
Don't know/refusal		13		2		26		29

*Source*: EWCS (Eurofound 2010a).

**Table A2:** Sample statistics by industry for the independent variables

		Production		High‐value services		Public administration		Accommodation and wholesale/retail	
	Range	Per cent/median	Std. dev.	Per cent/median	Std. dev.	Per cent/median	Std. dev.	Per cent/median	Std. dev.
Employee representative		63		50		68		34	
Involvement in improving work organization	1–5	3.07	1.46	3.3	1.33	3.31	1.37	2.93	1.47
Manager support	1–5	3.52	1.25	3.68	1.14	3.68	1.18	3.63	1.22
Female supervisor		14		23		27		28	
*Skills*									
High general skills		32		66		48		16	
Specific skills		45		2		5		12	
Low general skills		24		33		47		71	
*Firm size (no. employees)*									
1–9		18		35		16		46	
10–49		27		30		29		34	
50–99		14		8		14		8	
100–499		27		15		25		9	
500+		14		12		17		3	
*Country*									
Belgium		17		14		19		18	
Denmark		7		5		4		3	
Germany		13		10		8		11	
Greece		3		2		6		5	
Spain		4		5		4		5	
France		11		14		15		17	
Ireland		4		5		3		5	
Italy		8		7		7		6	
Luxembourg		2		10		4		4	
The Netherlands		5		5		5		3	
Austria		6		4		3		6	
Portugal		6		2		4		4	
Finland		5		5		2		4	
Sweden		5		6		6		2	
UK		5		6		9		7	
Female		32		54		46		55	
Age		41.82	11.21	40.53	11.03	44.16	10.42	37.28	12.07
Children <6 years		16		17		15		15	
Part‐time		13		19		20		32	
*Income*									
Low		39		32		36		32	
Middle		24		25		24		30	
High		25		27		25		23	
Don't know/refusal		12		15		16		15

*Source*: EWCS (Eurofound 2010a).

## References

[spol12270-bib-0001] Addabbo, T. , Giovannini, D. and Mazzucchelli, S. (2014), Italy country note. In P. Moss (ed.) International Review of Leave Policies and Research 2014, http://www.leavenetwork.org/lp_and_r_reports (accessed 28 April 2015).

[spol12270-bib-0002] Amable, B. (2003), The Diversity of Modern Capitalism, Oxford: Oxford University Press.

[spol12270-bib-0003] Berg, P. , Appelbaum, E. , Bailey, T. and Kalleberg, A. L. (2004), Contesting time: International comparisons of employee control of working time, Industrial & Labor Relations Review, 57, 3: 331–49.

[spol12270-bib-0004] Berg, P. , Kossek, E. E. , Misra, K. and Belman, D. (2014), Work‐life flexibility policies: Do unions affect employee access and use? Industrial & Labor Relations Review, 67, 1: 111–37.

[spol12270-bib-0005] Bloksgaard, L. and Rostgaard, T. (2014), Denmark country note. In P. Moss (ed.), International Review of Leave Policies and Research 2014, http://www.leavenetwork.org/lp_and_r_reports (accessed 28 April 2015).

[spol12270-bib-0006] Bonoli, G. (1997), Classifying welfare states: A two‐dimension approach, Journal of Social Policy, 26, 3: 351–72.

[spol12270-bib-0007] Braun, J. and Specht, M. (2009), Ergebnisse der ISW‐Betriebsrätebefragung 2008, WISO, 32, 2: 103–24.

[spol12270-bib-0008] Bridgen, P. and Meyer, T. (2005), When do benevolent capitalists change their mind? Explaining the retrenchment of defined‐benefit pensions in Britain *,* Social Policy & Administration, 39, 7: 764–85.

[spol12270-bib-0009] Budd, J. and Mumford, K. (2004), Trade unions and family‐friendly policies in Britain, Industrial and Labor Review, 57, 2: 204–22.

[spol12270-bib-0010] Chung, H . (2008), Do institutions matter? Explaining the use of working time flexibility arrangements of companies across 21 European countries using a multilevel model focusing on country level determinants *,* WZB Discussion Paper No. SP I 2008–07, Berlin: Berlin Social Science Center (WZB).

[spol12270-bib-0011] Chung, H. (2009), Flexibility for Whom? Working Time Flexibility Practices of European Companies, Ridderkerk: Ridderprint.

[spol12270-bib-0012] Chung, H. and Tijdens, K. (2013), Working time flexibility components and working time regimes in Europe: Using company‐level data across 21 countries, The International Journal of Human Resource Management, 24, 7: 1418–34.

[spol12270-bib-0013] Den Dulk, L. (2001), Work‐Family Arrangements in Organisations: A Cross‐National Study in the Netherlands, Italy, the United Kingdom and Sweden, Amsterdam: Rozenberg.

[spol12270-bib-0014] Den Dulk, L. , Peper, B. , Nevenka, C. S. , Lewis, S. , Smithson, J. and van Doorne‐Huiskes, A. (2011), Work, family, and managerial attitudes and practices in the European workplace: Comparing Dutch, British, and Slovenian financial sector managers, Social Politics, 18, 2: 300–29. 21966700 10.1093/sp/jxr009

[spol12270-bib-0015] Den Dulk, L. , Peters, P. and Poutsma, E. (2012), Variations in adoption of workplace work–family arrangements in Europe: The influence of welfare‐state regime and organizational characteristics, International Journal of Human Resource Management, 23, 13: 2785–808.

[spol12270-bib-0016] DiMaggio, P. I. and Powell, W. W. (1983), The iron cage revisited: Institutional isomorphism and collective rationality in organizational fields, American Sociological Review, 48, 2: 147–60.

[spol12270-bib-0017] Ebbinghaus B. (ed.) (2011) The Varieties of Pension Governance: Pension Privatization in Europe, Oxford: Oxford University Press.

[spol12270-bib-0018] Esping‐Andersen, G. , Gallie, D. , Hemerijk, A. and Myers, J. (2002), Why We Need a New Welfare State, New York, NY: Oxford University Press.

[spol12270-bib-0019] Estevez‐Abe, M. , Iversen, T. and Soskice, D. (2001), Social protection and the formation of skills: A reinterpretation of the welfare state In Hall P. A. and Soskice D. (eds), Varieties of Capitalism – The Institutional Foundations of Comparative Advantage, Oxford and New York, NY: Oxford University Press, pp. 145–83.

[spol12270-bib-0020] Eurofound (ed.) (2010a), 5th European Working Conditions Survey, 2010 – Sampling Implementation Report, Dublin: The European Foundation for the Improvement of Living and Working Conditions.

[spol12270-bib-0021] Eurofound (ed.) (2010b), Flexibility Profiles of European Companies – European Company Survey 2009, Dublin: The European Foundation for the Improvement of Living and Working Conditions.

[spol12270-bib-0022] European Commission (2009), Reconciliation between Work, Private and Family Life in the European Union, Luxembourg: Office for Official Publications of the European Communities.

[spol12270-bib-0023] Evans, J. M. (2002), Work/family reconciliation, gender wage equity and occupational segregation: The role of firms and public policy, Canadian Public Policy/Analyse de Politiques, 28, 1: 187–216.

[spol12270-bib-0024] Ferrera, M. (1996), The ‘Southern model’ of welfare in social Europe, Journal of European Social Policy, 6, 1: 17–37.

[spol12270-bib-0025] Figart, D. M. and Mutari, E. (2000), Work time regimes in Europe: Can flexibility and gender equity coexist? Journal of Economic Issues, 34, 4: 847–71.

[spol12270-bib-0026] Fleckenstein, T. and Seeleib‐Kaiser, M. (2011), Cross‐national perspectives on firm‐level family policies: Britain, Germany, and the US compared In Clasen J. (ed.), Converging Worlds of Welfare? British and German Social Policy in the 21st Century, Oxford: Oxford University Press, pp. 129–54.

[spol12270-bib-0027] Fleckenstein, T. , Saunders, A. M. and Seeleib‐Kaiser, M. (2011), The dual transformation of social protection and human capital comparing Britain and Germany, Comparative Political Studies, 44, 12: 1622–50.

[spol12270-bib-0028] Gerhartinger, P. , Specht, M. and Braun, J. (2010), Ergebnisse der ISW‐Betriebsrätebefragung 2010, WISO, 33, 4: 115–34.

[spol12270-bib-0029] Gregory, A. and Milner, S. (2009), Trade unions and work‐life balance: Changing times in France and the UK? British Journal of Industrial Relations, 47, 1: 122–46.

[spol12270-bib-0030] Hall P. A. and Soskice D. (eds) (2001), Varieties of Capitalism – The Institutional Foundations of Comparative Advantage, Oxford and New York, NY: Oxford University Press.

[spol12270-bib-0031] Hancké B., Rhodes M. and Thatcher M. (eds) (2007), Beyond Varieties of Capitalism: Conflict, Contradictions, and Complementarities in the European Economy, Oxford: Oxford University Press.

[spol12270-bib-0032] Heywood, J. S. and Jirjahn, U. (2009), Family‐friendly practices and worker representation in Germany, Industrial Relations: A Journal of Economy and Society, 48, 1: 121–45.

[spol12270-bib-0033] Ilsøe, A. (2012), The flip side of organized decentralization: Company‐level bargaining in Denmark, British Journal of Industrial Relations, 50, 4: 760–81.

[spol12270-bib-0034] Iversen, T. (2005), Capitalism, Democracy, and Welfare, Cambridge: Cambridge University Press.

[spol12270-bib-0035] Mahoney, J. and Goertz, G. (2006), A tale of two cultures: Contrasting quantitative and qualitative research, Political Analysis, 14, 3: 227–49.

[spol12270-bib-0036] Mares, I. (2001), Firms and the welfare state: When, why, and how does social policy matter to employers? In Hall P. A. and Soskice D. (eds), Varieties of Capitalism – The Institutional Foundations of Comparative Advantage, Oxford and New York, NY: Oxford University Press, pp. 184–212.

[spol12270-bib-0037] Milliken, F. J. , Martins, L. L. and Morgan, H. (1998), Explaining organizational responsiveness to work‐family issues: The role of human resource executives as issue interpreters, Academy of Management Journal, 41, 5: 580–92.

[spol12270-bib-0038] Mischke, M. (2011), Types of public family support: A cluster analysis of 15 European countries, Journal of Comparative Policy Analysis, 13, 4: 443–56.

[spol12270-bib-0039] Mood, C. (2010), Logistic regression: Why we cannot do what we think we can do, and what we can do about it, European Sociological Review, 26, 1: 67–82.

[spol12270-bib-0040] Morel N., Palier B. and Palme J. (eds) (2012), Towards a Social Investment Welfare State? Ideas, Policies and Challenges, Bristol: Policy Press.

[spol12270-bib-0041] O'Brien, M. , Moss, P. , Koslowski, A. and Daly, M. (2014), United Kingdom country note In Moss P. (ed.), International Review of Leave Policies and Research 2014, http://www.leavenetwork.org/lp_and_r_reports (accessed 28 April 2015).

[spol12270-bib-0042] Possenriede, D. and Plantenga, J. (2014), Temporal and locational flexibility of work, working‐time‐fit, and job satisfaction, IZA Discussion Paper No. 8436, Bonn: Institute for the Study of Labor (IZA).

[spol12270-bib-0043] Ravenswood, K. and Markey, R. (2011), The role of unions in achieving a family‐friendly workplace, Journal of Industrial Relations, 53, 4: 486–503.

[spol12270-bib-0044] Rigby, M. and O'Brien‐Smith, F. (2010), Trade union interventions in work‐life balance, Work, Employment and Society, 24, 2: 203–20.

[spol12270-bib-0045] Rille‐Pfeiffer, C. and Dearing, H. (2014), Austria country note. In P. Moss (ed.), International Review of Leave Policies and Research *2014*, http://www.leavenetwork.org/lp_and_r_reports (accessed 28 April 2015).

[spol12270-bib-0046] Russell, H. , O'Connell, P. J. and McGinnity, F. (2009), The impact of flexible working arrangements on work–life conflict and work pressure in Ireland, Gender, Work and Organization, 16, 1: 73–97.

[spol12270-bib-0047] Seeleib‐Kaiser, M. and Fleckenstein, T. (2009), The political economy of occupational family policies: Comparing workplaces in Britain and Germany, British Journal of Industrial Relations, 47, 4: 741–64.

[spol12270-bib-0048] Seeleib‐Kaiser, M. , Saunders, A. M. and Naczyk, M. (2012), Shifting the public‐private mix: A new dualisation of welfare? In Emmenegger P., Häusermann S., Palier B. and Seeleib‐Kaiser M. (eds), The Age of Dualization. The Changing Face of Inequality in Deindustrializing Societies, New York, NY: Oxford University Press, pp. 151–75.

[spol12270-bib-0049] Visser, J. (2013), ICTWSS: Database on Institutional Characteristics of Trade Unions, Wage Setting, State Intervention and Social Pacts in 34 Countries between 1960 and 2012, Amsterdam: AIAS.

[spol12270-bib-0050] Wood, S. J. , De Menezes, L. M. and Lasaosa, A. (2003), Family‐friendly management in Great Britain: Testing various perspectives, Industrial Relations, 42, 2: 221–50.

